# Characterization of Luminescent Materials with ^151^Eu Mössbauer Spectroscopy

**DOI:** 10.3390/ma11050828

**Published:** 2018-05-17

**Authors:** Franziska Steudel, Jacqueline A. Johnson, Charles E. Johnson, Stefan Schweizer

**Affiliations:** 1 Fraunhofer Application Center for Inorganic Phosphors, Branch Lab of Fraunhofer Institute for Microstructure of Materials and Systems IMWS, Lübecker Ring 2, 59494 Soest, Germany; franziska.steudel@imws.fraunhofer.de; 2 University of Tennessee Space Institute, Tullahoma, TN 37388, USA; jjohnson@utsi.edu (J.A.J.); cjohnson@utsi.edu (C.E.J.); 3 Faculty of Electrical Engineering, South Westphalia University of Applied Sciences, Lübecker Ring 2, 59494 Soest, Germany

**Keywords:** Mössbauer, Eu^2+^/Eu^3+^, luminescence

## Abstract

The application of Mössbauer spectroscopy to luminescent materials is described. Many solids doped with europium are luminescent, i.e., when irradiated with light they emit light of a longer wavelength. These materials therefore have practical applications in tuning the light output of devices like light emitting diodes. The optical properties are very different for the two possible valence states Eu${}^{2+}$ and Eu${}^{3+}$, the former producing ultraviolet/visible light that shifts from violet to red depending on the host and the latter red light, so it is important to have a knowledge of their behavior in a sample environment. Photoluminescence spectra cannot give a quantitative analysis of Eu${}^{2+}$ and Eu${}^{3+}$ ions. Mössbauer spectroscopy, however, is more powerful and gives a separate spectrum for each oxidation state enabling the relative amount present to be estimated. The oxidation state can be identified from its isomer shift which is between −12 and −15 mm/s for Eu${}^{2+}$ compared to around 0 mm/s for Eu${}^{3+}$. Furthermore, within each oxidation state, there are changes depending on the ligands attached to the europium: the shift is more positive for increased covalency of the bonding ligand X, or Eu concentration, and decreases for increasing Eu–X bond length.

## 1. Introduction

In the last half of the 20th century, solid state physics has been responsible for a remarkable revolution in electronics with the replacement of the vacuum tube by semiconductor transistors. Not quite so dramatic but more visible, literally, is the replacement of incandescent lighting by light emitting diodes (LEDs) with luminescent materials. Among these materials are the lanthanides, which have unique magnetic, luminescent, and electrochemical properties and are therefore used for many different applications, such as magnets, batteries, superconductors or in optical devices. In particular, europium has attracted much attention due to its red emission in the trivalent state (Eu${}^{3+}$), which is widely used in fluorescent lamps, displays, and recently for solid-state lighting [1,2]. The broad emission of divalent europium (Eu${}^{2+}$) is used in lighting applications [3] as well as euro bank notes [4].

As a rule when substituting for trivalent ions (Al${}^{3+}$, Y${}^{3+}$, La${}^{3+}$) europium generally forms Eu${}^{3+}$, while for divalent ions (Mg${}^{2+}$, Ca${}^{2+}$, Sr${}^{2+}$, Ba${}^{2+}$), it goes in as Eu${}^{2+}$. In some cases, the optical activation with divalent europium turns out to be difficult, since Eu${}^{2+}$ is easily oxidized to Eu${}^{3+}$. An exact knowledge of the Eu${}^{2+}$ and Eu${}^{3+}$ content is necessary to guarantee an efficient light output and to optimize the material for practical applications. Eu${}^{2+}$ and Eu${}^{3+}$ are clearly distinguishable by photoluminescence spectroscopy. Eu${}^{2+}$ is characterized by a broad emission, while Eu${}^{3+}$ shows narrow emission lines. Both Eu${}^{2+}$ and Eu${}^{3+}$ are sensitive to changes in the surrounding crystal field. Photoluminescence spectroscopy, however, does not allow a quantitative analysis of Eu${}^{2+}$ and Eu${}^{3+}$ ions. For example, Eu${}^{2+}$ does not fluoresce in the fluorozirconate base glass, although it is clear from electron paramagnetic resonance (EPR) [5] and Mössbauer studies [6] that it is present in the glass.

X-ray absorption spectroscopy is one method to determine not only the charge of the doped europium ions, but also allows a quantitative analysis [7,8]. However, it requires access to synchrotron radiation sources.

${}^{151}$Eu Mössbauer spectroscopy has been established as a sensitive tool to distinguish quantitatively between Eu${}^{2+}$ and Eu${}^{3+}$ ions and to investigate the local structure around europium ions in solids. Unlike electron paramagnetic resonance (EPR) which cannot be used for trivalent europium ions (since the ${}^{7}$F${}_{0}$ ground state is not paramagnetic), information about both oxidation states emerges directly from the Mössbauer isomer shift.

The Mössbauer Effect is the recoilless resonant fluorescence of gamma-radiation. It was discovered in ${}^{191}$Ir by Rudolf L. Mößbauer in 1958 [9] for which he received the Nobel Prize in 1961. After it was observed in ${}^{57}$Fe, the field developed so fast that the first International Mössbauer Conference took place in 1960 at the University of Illinois [10]. Since then, the Mössbauer Effect was found in many isotopes including ${}^{119}$Sn, ${}^{121}$Sb, and ${}^{151}$Eu and has been applied in many fields of sciences, such as physics, chemistry, biology, and medicine. A review of ${}^{151}$Eu work has been written by Grandjean and Long [11]. An index of Mössbauer data is available from the Mössbauer Effect Data Center [12].

Many review papers assessing Mössbauer spectroscopy as a characterization technique for a wide variety of materials have been published. A general review paper has been published in 1968 [13], followed by Mössbauer spectroscopy on iron [14], glass [15,16,17], Fe/S proteins [18], and europium chalcogenides [19] among others. No review paper is known for the investigation of europium luminescence using Mössbauer spectroscopy. In this article, its application to europium-containing inorganic luminescent materials is reviewed.

## 2. The Lanthanide Ions Eu${}^{2+}$/Eu${}^{3+}$ and Their Optical Properties

**Figure 1:**
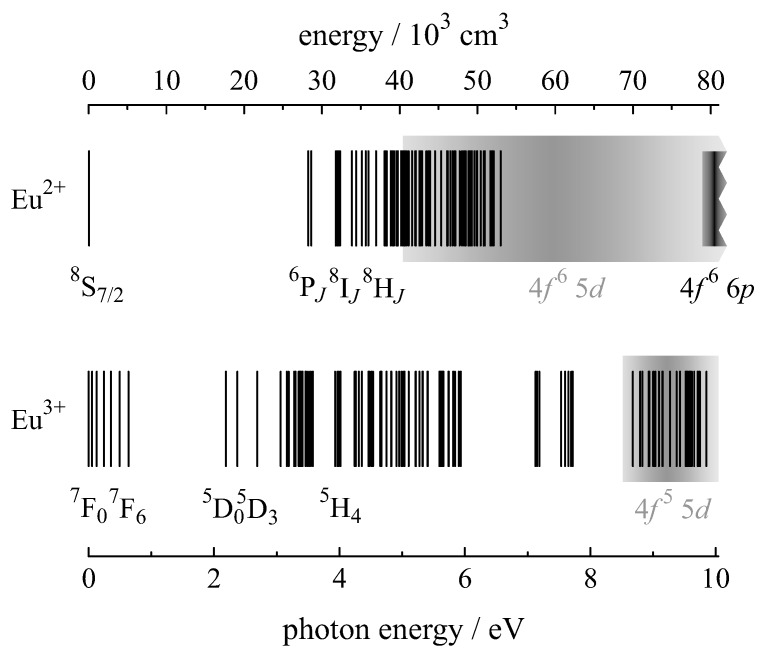
Energy level diagrams of Eu${}^{2+}$ (**top**) and Eu${}^{3+}$ (**bottom**). The 4$f^{n}$ levels are depicted as solid black lines and the closely spaced levels of 4$f^{n-1}$ 5*d* and 4$f^{n-1}$ 5*p* are depicted as grey bands [20,21,22].

Europium is the chemical element with the atomic number 63 and the electron configuration [Xe] 4$f^{7}$6$s^{2}$. As for all lanthanides, the most stable oxidation state is +3, but europium forms divalent compounds as well. Divalent europium is rather unstable and oxidizes in air to form trivalent Eu compounds. Both Eu${}^{2+}$ and Eu${}^{3+}$ have an incompletely filled 4f shell, which is shielded by the filled 5s and 5p shells. This peculiar electronic configuration is responsible for their unique optical and luminescent behaviour. The energy level diagrams for Eu${}^{2+}$ and Eu${}^{3+}$ are shown in Figure 1. Due to the shielding, $4f^{n}$ energy levels are only weakly influenced by the host material and can be depicted as solid lines each with a characteristic energy. Using the term symbol ${}^{2S+1}L_{J}$ where *S* is the total spin quantum number, *L* is the total orbital quantum number, and *J* is the total angular momentum quantum number the ground state configuration for Eu${}^{2+}$ and Eu${}^{3+}$ are ${}^{8}$S${}_{7/2}$ and ${}^{7}$F${}_{0}$, respectively.

Intra-configurational 4f→4f transitions are forbidden by the parity selection rule. However, the 4f wave function mixes with an opposite parity wave function, e.g., 5d, and the $4f^{n}$ transitions gain intensity. The inter-configurational 4f→5d transitions are allowed and appear in the spectra as broad absorption and emission bands; they are depicted as grey bands in Figure 1. Additionally, their energy depends strongly on the composition of the host structure.

Whereas the red luminescence of trivalent europium results from forbidden 4f→4f transitions, the luminescence of divalent europium results from allowed 4f→5d transitions. Both are discussed in more detail in the following sections.

### 2.1. Optical Properties of Eu${}^{2+}$

#### 2.1.1. Eu${}^{2+}$ in Luminescent Fluoride and Oxide Glasses

ZBLAN glasses were discovered accidentally in 1975 by Poulain and Lucas [23] at the University of Rennes in France. They were typically used for optical fibers due to their high infrared transmittance [24,25]. ZBLAN glass, however, is fragile and sensitive to acids. Interestingly, its manufacture has been initiated on the International Space Station to avoid defects [26]. In 2003, Schweizer et al. thought of substituting some of the BaF${}_{2}$ for BaCl${}_{2}$ and adding Eu${}^{2+}$ [27]. On heating, the BaCl${}_{2}$ drops out as a crystallite, incorporating the optically active divalent europium. This initiated an investigation into their suitability as X-ray image plates for medical diagnosis [28]. Further applications of fluorozirconate glass are optical devices, such as colour displays [29] or up-converting and down-converting glass layers for solar cells [30,31]. Glass ceramics with hexagonal phase crystallites can be used as scintillators [32] while the orthorhombic phase is suitable for storage phosphors [33].

**Figure 2:**
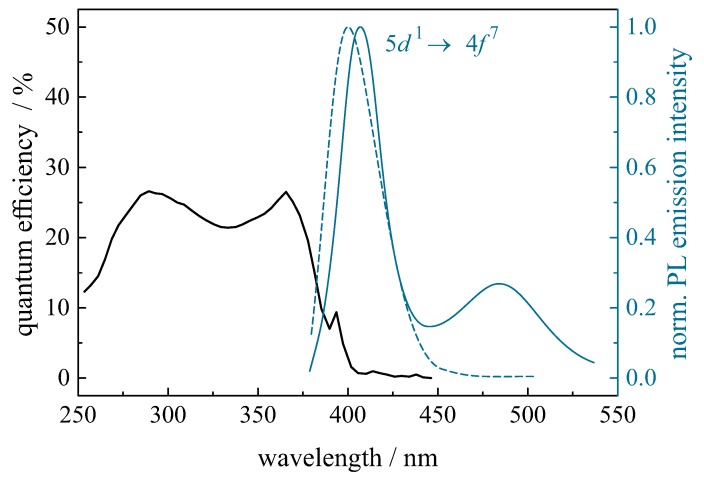
Normalized PL emission spectra of Eu${}^{2+}$-doped fluorochlorozirconate glass processed at 260 ${}^{\circ }$C (solid curve) and 290 ${}^{\circ }$C (dashed curve) for 20 min. The PL was excited at 285 nm and recorded at room temperature. The 260 ${}^{\circ }$C processed sample only contains hexagonal BaCl${}_{2}$ in a glass matrix, while the sample processed at 290 ${}^{\circ }$C contains mainly orthorhombic BaCl${}_{2}$ in a glass matrix [34]. The quantum efficiency spectrum (on the left side) has been recorded for the 290 ${}^{\circ }$C processed sample.

Europium can be incorporated in its divalent and trivalent state in ZBLAN glass. Eu${}^{3+}$ shows its typical emissions in the red spectral range (see Section 2.2) in the fluorozirconate base glass. Upon annealing, some of the Eu${}^{2+}$ ions are incorporated into the BaCl${}_{2}$ nanocrystals leading to an intense Eu${}^{2+}$-related fluorescence under ultraviolet excitation. After 20 min of annealing at 260 ${}^{\circ }$C the Eu${}^{2+}$ fluorescence spectrum shows a main emission band at 407 nm (Figure 2, solid curve) and a weaker, but broader emission at 485 nm. After annealing at 290 ${}^{\circ }$C (Figure 2, dashed curve), the 407 nm emission is shifted to 402 nm, while the 485 nm band has completely disappeared. The 402-nm and 407-nm emission bands are attributed to Eu${}^{2+}$ in hexagonal and orthorhombic BaCl${}_{2}$, respectively, while the origin of the additional weaker but broader band at 485 nm is unknown. These observations are described in detail in [34]. The quantum efficiency spectrum of Eu${}^{2+}$ in ZBLAN containing orthorhombic BaCl${}_{2}$ crystallites is shown on the left.

Oxide glasses containing divalent lanthanide ions have attracted considerable attention as optical and magneto-optical devices. These glasses are advantageous for applications, such as frequency and time domain optical memories. Aluminoborate glasses containing a large amount of Eu${}^{2+}$ show a Faraday effect [35]. The paramagnetic Faraday effect can be used for magneto-optical devices, such as optical isolators, optical switches, and optical shutters [36].

#### 2.1.2. Eu${}^{2+}$ in (Persistent) Phosphors/Aluminates

Persistent luminescence (also known as phosphorescence) is a phenomenon in which light (UV, visible or IR) is emitted for minutes, hours or even days after the initial excitation. The mechanism underlying this phenomenon is not fully understood but is known to involve energy traps. These traps are filled during excitation. After excitation, the stored energy is released to emitter centres, which gradually emit the light. There are many persistent phosphors, but the most studied are the strontium aluminates doped with Eu${}^{2+}$ and Dy${}^{3+}$ due to their high brightness, long lifetime, and stability. The slow decay gives the ion the ability to store information, which may be read later by optical (laser) or thermal stimulation [37]. However, large-scale production of these materials needs to be developed for them to realize their full commercial potential. For a detailed review of persistent phosphors, the reader should look at the following references [38,39].

BaMgAl${}_{10}$O${}_{17}$:Eu${}^{2+}$ has two broad excitation bands at approximately 270 nm and 300 nm and shows a blue luminescence with the peak near 450 nm. Together with a red-emitting phosphor and a green-emitting phosphor, it yields a white emitting blend for fluorescent lamps and plasma display panels [40,41]. However, it is unstable in a variety of lamp-related processing conditions and also during the lamp life.

Eu${}^{2+}$ in other aluminates and alumosilicates shows persistent luminescence, e.g., CaAl${}_{2}$O${}_{4}$:Eu${}^{2+}$ (blue) [42], SrAl${}_{2}$O${}_{4}$:Eu${}^{2+}$ (green) [43], Sr${}_{4}$Al${}_{14}$O${}_{25}$:Eu${}^{2+}$ (blue) [44], and CaAl${}_{2}$Si${}_{2}$O${}_{8}$:Eu${}^{2+}$ (white) [45]. These compounds have applications to thermoluminescence, used for dating and radiation monitoring [46].

#### 2.1.3. Eu${}^{2+}$ in Other Luminescent Materials

CaS-based phosphors are studied for dosimetry applications [47]. For Eu${}^{2+}$-doped CaS containing additional impurities (such as Sm${}^{2+}$), the stored dose can be read out upon optical stimulation in the infrared spectral range (in a so-called optically stimulated luminescence (OSL) process) instead of thermoluminescence [48].

Nitridosilicates provide efficient luminescent materials that are industrially applied in commercial phosphor-converted LEDs [49,50]. Here, Eu${}_{2}$SiN${}_{3}$ is of particular interest since it is the only mixed-valence europium nitridosilicate, i.e., it has two different crystallographic Eu sites at which one site is occupied by Eu${}^{2+}$ ions, while the other site is occupied by Eu${}^{3+}$ ions [51]. It is also noteworthy that Eu${}_{2}$SiN${}_{3}$ has a black colour due to its small band gap of 0.2 eV [50].

Oxonitridosilicates combine structural features and properties of both, oxosilicates and nitridosilicates. EuSi${}_{2}$O${}_{2}$N${}_{2}$ shows a narrow yellow emission and is investigated for 2- and 3-phosphor-converted light-emitting diodes [52].

### 2.2. Optical Properties of Eu${}^{3+}$

Eu${}^{3+}$ shows in different materials an efficient red luminescence with high quantum efficiencies. In 1989, the quantum efficiency of Eu${}^{3+}$ in BaCa${}_{2}$Y${}_{6}$O${}_{12}$ was found to be up to 25% [53]. The quantum efficiency of nanocrystalline powder of Lu${}_{2}$O${}_{3}$:Eu${}^{3+}$ reaches 90% [54] and that of bulk Y${}_{2}$O${}_{3}$:Eu${}^{3+}$ of 92% [55]. In borate and in fluorozirconate glass, the Eu${}^{3+}$ quantum efficiency was determined to be 86% [56] and 94% [57], respectively.

#### 2.2.1. Eu${}^{3+}$ in Luminescent Glasses

Eu${}^{3+}$ was used in fibre lasers in the early 1990s, mainly as a co-dopant with other rare earths to enhance efficiency [58,59]. More recently, it has been used as a down-converter in photovoltaic application [60,61] and to produce white light in LED’s, also as a co-dopant [56,57,62]. Energy transfer is the key to increase efficiency in both cases, the details of which are discussed within the references given here. The literature on Eu${}^{3+}$-doping of borate glasses is more plentiful than that involving ZBLAN. Again, recent research has focused on the creation of white light and many glasses are not pure borates but combinations, such as fluoroborate, borogermanate, aluminoborate, lead borate, and borosilicate, for example. Whatever the host, Eu${}^{3+}$ is responsible for the red component of emission and other rare earths complement Eu${}^{3+}$ in order to emit other colours, although white light is the most desirable right now. Older papers, starting in the early 1990s, focused on the study of fundamental luminescence properties.

**Figure 3:**
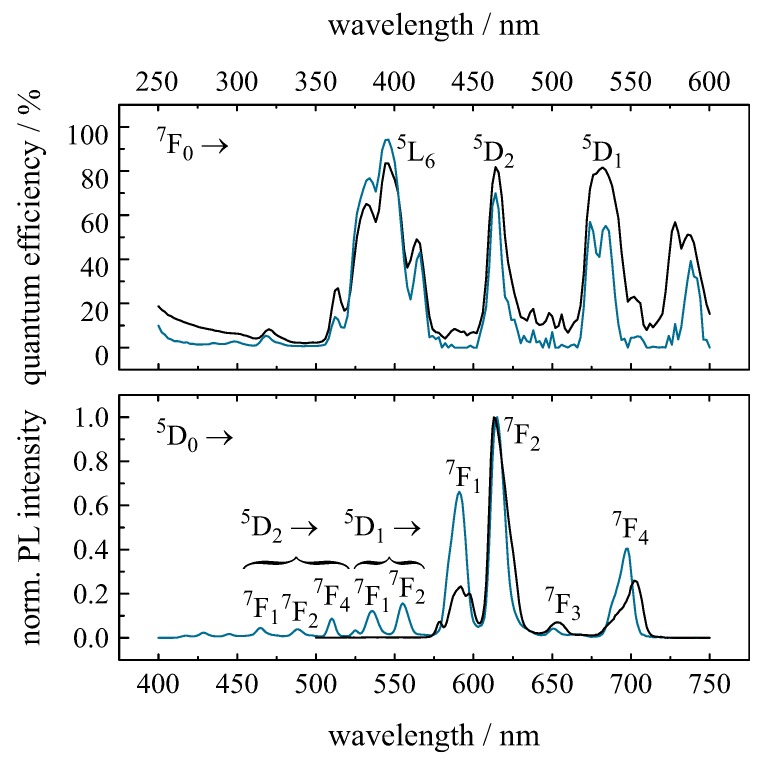
Absolute PL quantum efficiency and PL emission spectra of Eu${}^{3+}$ single-doped borate (black curves) and ZBLAN glass (blue curves). The Eu${}^{3+}$ emissions are recorded under 395-nm excitation at room temperature [57].

The absolute PL quantum efficiency (QE) and PL emission spectra of Eu${}^{3+}$-doped borate (66B${}_{2}$O${}_{3}$·33BaO·1Eu${}_{2}$O${}_{3}$, values in mol %) and ZBLAN glass (51ZrF${}_{4}$·20BaF${}_{2}$·20NaF·3.5LaF${}_{3}$·3AlF${}_{3}$·0.5InF${}_{3}$·2EuF${}_{3}$, values in mol %) are depicted in Figure 3. The maximum quantum efficiency value is found in ZBLAN glass and amounts to 94% for 395-nm excitation (${}^{7}$F${}_{0}$ to ${}^{5}$L${}_{6}$ transition). The glasses can also be excited in the blue spectral range, for instance at 465 nm (${}^{7}$F${}_{0}$ to ${}^{5}$D${}_{2}$ transition), resulting in QE values of 70% and 82% for ZBLAN and borate glass, respectively. In comparison to ZBLAN glass, borate glass provides a higher QE in the longer wavelength range, but a lower one in the short wavelength range.

In both glass systems, transitions from the excited state, ${}^{5}$D${}_{0}$, to the ground state levels, ${}^{7}$F${}_{J}$ (J=1,2,3,4,5, and 6) are observed leading to the typical emission in the red spectral range. In ZBLAN glass, additional emissions in the ultraviolet/blue spectral range are observed originating from the excited states, ${}^{5}$D${}_{1}$ and ${}^{5}$D${}_{2}$, to the ground states, ${}^{7}$F${}_{J}$ (J=0,1,2, and 4). In borate glass, emissions from the excited states, ${}^{5}$D${}_{1}$ and ${}^{5}$D${}_{2}$, are quenched due non-radiative relaxation, whereas in ZBLAN glass the probability for radiative emissions from these levels is significantly higher. The ${}^{5}$D${}_{0}$ and ${}^{5}$D${}_{1}$ states are separated by 1750 cm${}^{-1}$ [20]. In the case of borate glass with a maximum phonon frequency of 1400 cm${}^{-1}$ [63,64], only one to two phonons are needed to bridge the gap, while, for ZBLAN glass, with a maximum phonon frequency of 580 cm${}^{-1}$ [65], more than three phonons are necessary. Thus, the non-radiative transition rates in ZBLAN glass are significantly smaller than in borate glass enabling radiative emission.

The electric-dipole transition ${}^{5}$D${}_{0}$ to ${}^{7}$F${}_{2}$ is hypersensitive to variations in crystal symmetry [1]. The high intensity of this transition in borate glass indicates the amorphous nature of the matrix material without inversion symmetry for the Eu${}^{3+}$ ion. For ZBLAN glass, the intensity of the ${}^{5}$D${}_{0}$ to ${}^{7}$F${}_{1}$ transition is higher than in borate glass, which implies a higher crystallinity of ZBLAN glass compared to borate glass. Therefore, Eu${}^{3+}$ is a useful spectroscopic probe of the environment surrounding the lanthanide ion.

#### 2.2.2. Eu${}^{3+}$ in Lanthanide Oxides

Eu-doped sesquioxides Ln${}_{2}$O${}_{3}$ (Ln = In, Sc, Y, La, Gd, Lu) are very important materials as they are used as the red-emitting phosphors in fluorescent lamps and colour television projection tubes [66]. Y${}_{2}$O${}_{3}$:Eu${}^{3+}$ nanoparticles in spherical morphology are used for flat-panel displays [67]. Lu${}_{2}$O${}_{3}$:Eu is a very attractive host for scintillators [68] or x-ray phosphors [69], due to a high density of lutetia, which ensures that ionizing radiation is efficiently absorbed in relatively thin layers of lutetia-based phosphors.

#### 2.2.3. Eu${}^{3+}$ in Other Luminescent Materials

The production of III-V semiconductor based LEDs with efficient emission in the green and red spectral range is still challenging. GaN, doped with erbium and europium, enables the emission in the green and red spectral range, respectively, for lighting applications of optoelectronic devices [70].

Pyrochlore materials are known for their good thermal properties. Lanthanide-doped pyrochlores are used for contact-free surface temperature measurements [71].

Europium-doped titanium dioxide is developed as a substitute for the high-cost red-emitting phosphor Y${}_{2}$O${}_{3}$:Eu. TiO${}_{2}$:Eu is demonstrated to be a good sensitizer to absorb light and transfer energy to Eu${}^{3+}$ ions [72,73]. It is also advantageous for practical applications due to its low cost, its chemical and thermal stability, and its good mechanical properties [72,74].

YVO${}_{4}$:Eu is a well-known red phosphor applied in cathode ray tubes, fluorescent lamps, and plasma displays. It provides high efficiency, colour purity, and thermal stability [75,76]. In addition, it is used as a fluorescent biological label for the detection of Na${}^{+}$ channel dynamics on cell membranes [77]. EuVO${}_{4}$ might be suitable to track biological systems, such as histidine and bovine serum albumin [78].

Zirconia is a widely-used material in optics due to its wide band-gap, high transparency, high refractive index and hardness [79]. Eu-doped ZrO${}_{2}$ has been investigated for lamp and display applications [80].

Recently, Eu-doped yttrium aluminium garnet (Y${}_{3}$Al${}_{5}$O${}_{12}$, YAG) gained increasing attention as phosphor in white LEDs and laser-active material [81,82]. It crystallizes in the cubic form [81].

## 3. Europium Mössbauer Spectroscopy

### 3.1. Mössbauer Isotope ^151^Eu

**Figure 4:**
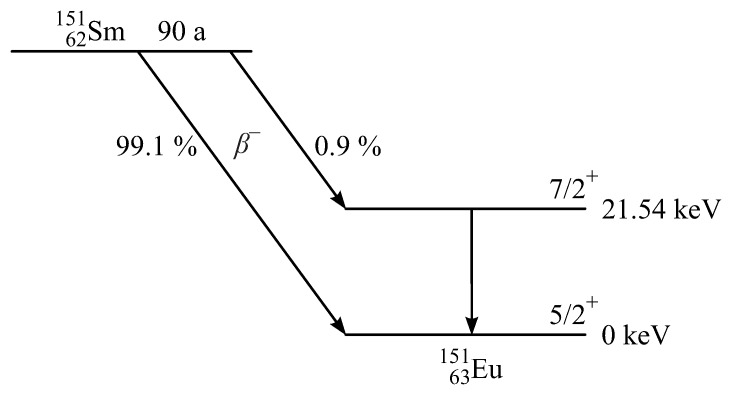
Nuclear decay scheme for ${}^{151}$Sm and ${}^{151}$Eu [83].

Eu has two naturally occurring isotopes, namely ${}^{151}$Eu (47.82%) and ${}^{153}$Eu (52.18%) of which the former is the most useful for Mössbauer spectroscopy owing to its low γ-ray energy of 21.54 keV. The source used is ${}^{151}$Sm, and its decay scheme is shown in Figure 4. The half-life of ${}^{151}$Sm with 90 years is very long [83]. Two different $\beta^{-}$-decays occur. Only 0.9% decays into the excited state of ${}^{151}$Eu. The remaining 99.1% goes to the ground state [83]. The 21.54 keV transition from the excited state with spin +7/2 to the ground state of ${}^{151}$Eu with spin +5/2 has a lifetime of 14 ns and a resulting linewidth of 47 neV. Mössbauer isotopes must have a long lifetime for the decay of the excited state and very low lying excited states. These criteria exclude some isotopes. ${}^{151}$Eu is the most used Mössbauer isotope of the lanthanide elements.

To produce a spectrum, the γ-ray energy is varied using the Doppler effect by moving the ${}^{151}$Sm source relative to the ${}^{151}$Eu-containing absorber. A plot of transmitted γ-ray counts against velocity yields the Mössbauer spectrum. The velocities needed are of the order of millimetres per second (in non-SI units this is a furlong per fortnight—a snail’s pace!). The linewidth is governed by the lifetime of the 21.54 keV transition and is 2.52 mm/s. The 21.54 keV radiation may also be produced with synchrotron radiation with an appropriate monochromator. Such facilities can be found at the European Synchrotron Radiation Facility (ESRF) in Grenoble (France), the Applied Photon Source (APS) at the Argonne National Laboratory (USA) or at the Super Photon ring (SPring-8) at the Harima Science Park (Japan).

Mössbauer measurements are usually performed in transmission geometry, but using backscattering geometry, γ-rays, x-rays, conversion electrons or Auger electrons may be detected as the excited nuclear state decays back to the ground state. For the ${}^{151}$Eu isotope, there are no conversion electrons [84,85], but Bibicu et al. [85] found that this isotope emits Auger electrons. The transmission geometry gives volume information, while the backscattering geometry gives predominantly surface information.

In Mössbauer spectra, three types of nuclear interactions are observable: (i) isomer (chemical) shift; (ii) quadrupole splitting; and (iii) magnetic (hyperfine) splitting.

### 3.2. Isomer Shift

The isomer shift arises from the electric monopole interaction between the nucleus and the surrounding electron shell. The overlap of the charge density distribution of *s*-electrons with the nucleus causes a shift of the energy levels of the nucleus. The shift is observable because the ground and excited nuclear states have different radii and hence different overlaps with the electron cloud. It cannot be measured directly and therefore is quoted relative to a known absorber. In this paper, the values are given with respect to EuF${}_{3}$. The isomer shift is a good indicator for the valence state of the isotope. It is useful for the investigation of valence states, ligand bonding states, and electron shielding.

Eu${}^{3+}$ compounds exhibit isomer shifts between 0 and +3 mm/s. The large difference in isomer shifts of about 12 mm/s between divalent and trivalent Eu compounds results mainly from the shielding effect of the additional 4f electron in Eu${}^{2+}$ compounds; Eu${}^{2+}$ has the configuration $4f^{7}$, while Eu${}^{3+}$ has $4f^{6}$. Other effects like bond lengths, covalency, and coordination numbers produce less pronounced variations up to ±2 mm/s for Eu${}^{3+}$ and ±1 mm/s for Eu${}^{2+}$ but are clearly resolved due to the large difference between the nuclear radii of the ground and the excited state for the ${}^{151}$Eu resonance, respectively. The isomer shifts of Eu in binary compounds are listed in Table 1. It is evident from Table 1 that fluorine is the most ionic ligand and therefore it is mentioned first in the following chapters.

### 3.3. Quadrupole Interaction

**Figure 5:**
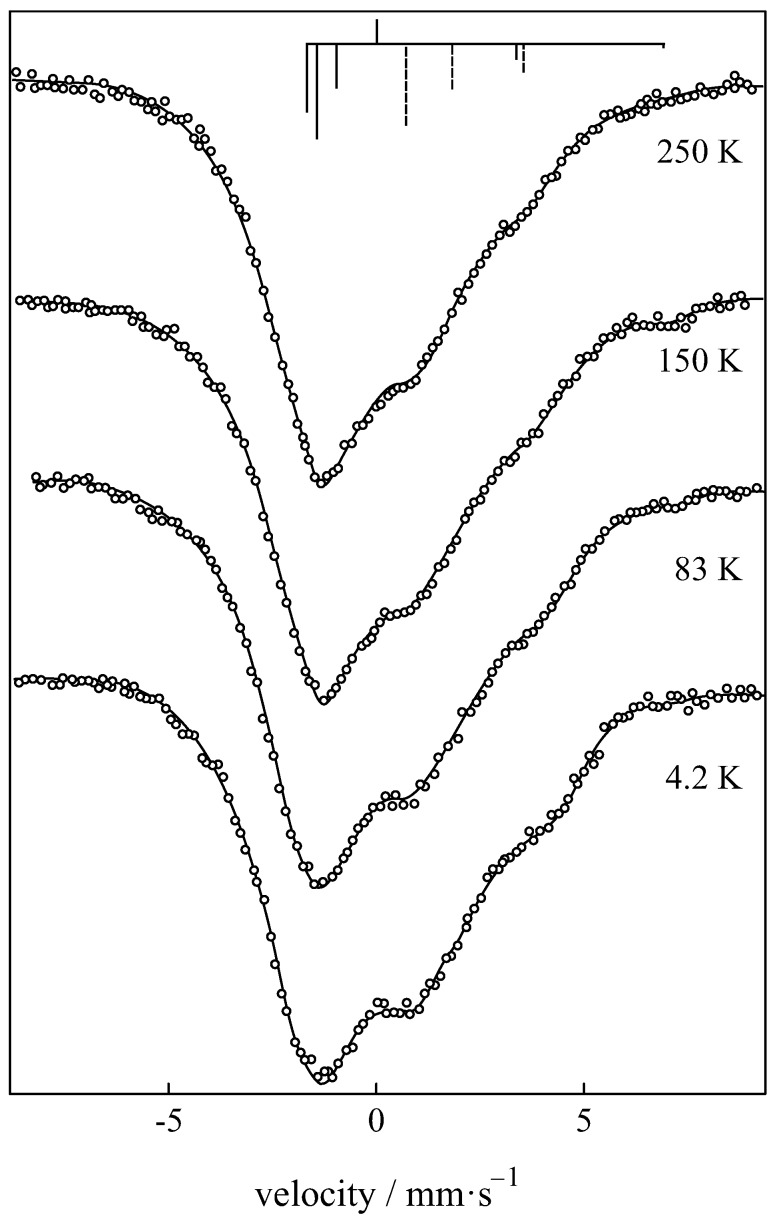
Mössbauer spectra of Eu${}_{2}$Ti${}_{2}$O${}_{7}$ showing quadrupole splitting [87].

Quadrupole splitting may arise for nuclei in states with an angular momentum quantum number I>1/2 due to their non-spherical charge distribution so that they have an electric quadrupole moment *Q*. This causes a split of the nuclear energy levels in the presence of an anisotropic electric field (anisotropic electronic charge distribution or ligand arrangement) when the lattice has a non-cubic structure. The charge distribution is characterized by the electric field gradient (EFG). From the quadrupole splitting, information on oxidation state, spin state, and site symmetry can be obtained. In a compound with cubic symmetry, the electric field gradient (and therefore the quadrupole interaction parameter) is zero and a single transition is observed [11], which is the case for the majority of the papers, summarized in this review article. If a threefold or fourfold axis is present, the electronic charge distribution will be symmetrical and eight transitions are allowed (see Figure 5). If there is no threefold or fourfold symmetry axis passing through the nucleus, the components of the electric field along the principal axes are different, the asymmetry parameter is non-zero, and there are 12 allowed transitions [86].

The quadrupole splitting is usually only partly resolved owing to the linewidth of about 2.5 mm/s. This gives an asymmetrically broadened line profile (see Figure 5), which for small interactions, has to be distinguished from overlapping contributions from more than one inequivalent site.

### 3.4. Magnetic Hyperfine Interaction

Magnetic hyperfine splitting is caused by the interaction between the nuclear magnetic moments and the magnetic field of the electrons on the atom. It is only observed for Eu${}^{2+}$ and to observe it requires magnetic dilution so that electron spin relaxation rates are slow. The paramagnetic hyperfine fields are typically of the order of 30 T.

**Figure 6:**
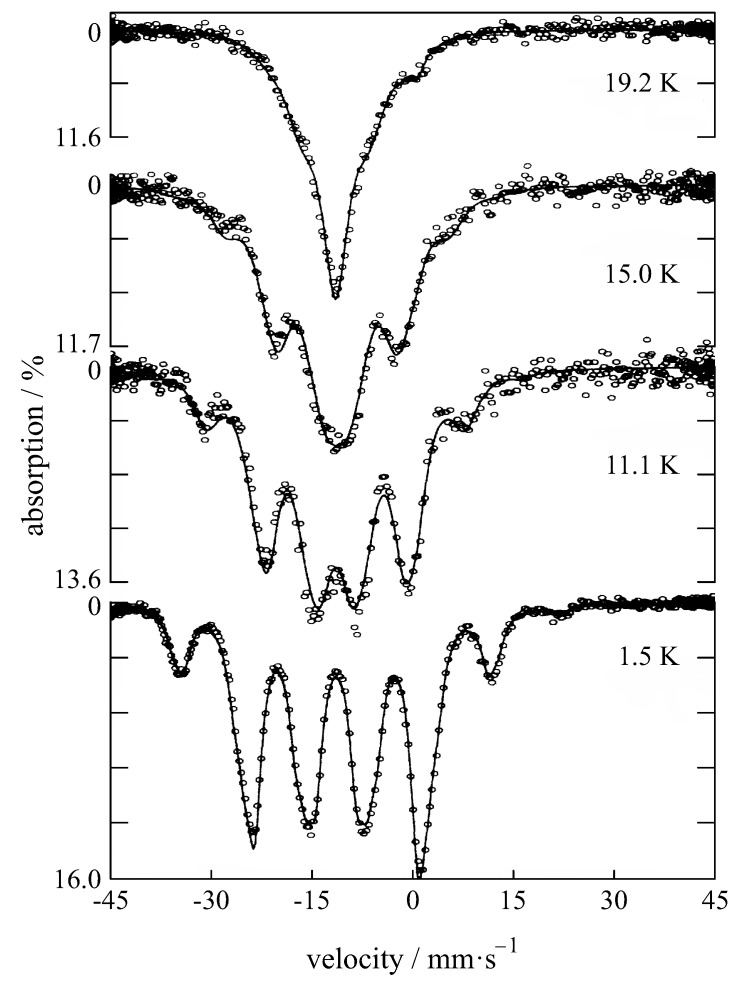
Mössbauer spectra of Eu${}_{0.65}$La${}_{0.35}$S showing magnetic hyperfine splitting arising from slow electron spin relaxation [88].

The nuclear spin *I* splits into 2I+1 sublevels, i.e., six levels for the ground state of ${}^{151}$Eu (I=5/2) and eight for the excited state (I=7/2). The selection rules Δm=0,±1 give rise to a symmetric 18-line spectrum, where, because of the smaller *g*-value of the excited state, it has the appearance of a six-line spectrum with broadened lines (see Figure 6).

### 3.5. Linewidth (FWHM)

The linewidth of the absorption line is dependent on the lifetime of the nuclear excited state [89]. In the case of an ideally thin absorber, the linewidth is twice the natural linewidth [89]. Usually, the linewidth of an amorphous material is broader than that of the corresponding crystalline material, e.g., Eu${}^{3+}$ in metaphosphate has a linewidth of 1.96 mm/s and 1.76 mm/s for the amorphous and crystalline material, respectively [90].

## 4. Materials Overview

An overview of different luminescent materials, containing Eu${}^{2+}$ and Eu${}^{3+}$ with the corresponding isomer shifts at room temperature, is given in Table 2 and Table 3, respectively. The isomer shift is in the range −9.7 to −14.3 mm/s for Eu${}^{2+}$ and −0.93 to +1.47 mm/s for Eu${}^{3+}$.

**Figure 7:**
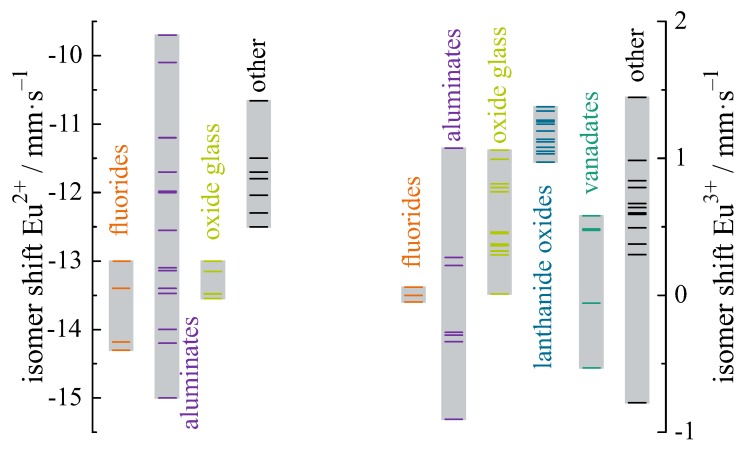
Plot of isomer shifts for the Eu compounds summarized in Table 2 and Table 3.

The isomer shifts listed in Table 2 and Table 3 are visualized in Figure 7, sorted for different material systems. Fluorides show isomer shifts from −14.3 to −13 mm/s and oxide glasses from −13.5 to −13 mm/s. Eu${}^{2+}$ in different kinds of aluminates have the largest range of isomer shifts from −9.7 to −15 mm/s. For materials, such as vanadates, sulphides, nitrides, and titanium dioxide, only a few papers were found and therefore depicted as “other” with isomer shifts ranging from −10.5 to −12.5 mm/s. For Eu${}^{3+}$, the isomer shifts vary from −1 to +1.4 mm/s with fluorides having isomer shifts at approximately 0 mm/s, because the isomer shifts in this paper are given relative to EuF${}_{3}$. Eu${}^{3+}$ has isomer shifts from 0 to 1 mm/s in oxide glasses and −0.6 to +0.6 mm/s in vanadates. In aluminates and other materials, the Eu${}^{3+}$ isomer shifts range from approximately −0.9 to +1 mm/s.

### 4.1. Fluorides

CaF${}_{2}$ with Eu${}^{2+}$ impurities occurs in nature as the mineral fluorite (or fluorspar) and the term fluorescence originates from its luminescent properties. Eu${}^{2+}$ in CaF${}_{2}$ shows strong luminescence, while that of Eu${}^{3+}$ is much weaker [128]. Implanted Eu${}^{2+}$ substituting for Ca${}^{2+}$ luminesces in the violet spectral range at 420 nm, while a second emission band at 680 nm arises from interstitial sites; the latter is eliminated on heating [129].

The ${}^{151}$Eu Mössbauer spectra of highly diluted (0.1 mol %) Eu${}^{2+}$ ions in CaF${}_{2}$ showed an almost temperature-independent asymmetrically split pattern, arising from the paramagnetic hyperfine interaction *A**S.I*** in a cubic crystal field with slow electron spin relaxation. In a small external magnetic field *B* of 0.2 T such that $g\mu_{B}B>A$, an almost symmetrical pattern was observed. Both, the spectra with and without an external field, are well described using the spin Hamiltonian and previous electron paramagnetic resonance data. A more concentrated (2 mol % Eu${}^{2+}$) sample exhibited a strongly broadened symmetrical resonance line due to an increased Eu–Eu spin relaxation rate in an external magnetic field of 0.2 T. The Mössbauer spectra exhibited further broadening and additional magnetic structures due to the reduced relaxation rate. When a large field of 6 T was applied such that $g\mu_{B}B$ is much larger than the crystal field splitting, a fully resolved hyperfine pattern was observed at 2.5 K, with an effective field at the Eu nuclei of −33.7 T; at higher temperatures, superimposed patterns originating from excited electronic states were observed in the spectra [92].

Fluorophosphate glasses with the composition (45−x)AlF${}_{3}$·*x*AlPO${}_{4}$·5EuF${}_{3}$·30CaF${}_{2}$·20BaF${}_{2}$ (x=0–20) showed only trivalent Eu [107]. The isomer shift increases slightly with increasing Eu concentration.

### 4.2. Fluorochlorozirconate (FCZ) Glasses

Much work has been done on fluorochlorozirconate (FCZ) glasses and glass ceramics. Coey et al. [93] made measurements on 61ZrF${}_{6}$·12BaF${}_{2}$·7ThF${}_{4}$ (values in mol %) doped with 20EuF${}_{2}$. Most of the europium was Eu${}^{2+}$ with an isomer shift of −14.18 mm/s corresponding to a large coordination number ranging between 8 and 12 which is typical for glasses. While the Eu${}^{2+}$ resonance line in the glass is extremely broad compared with EuF${}_{2}$, the Eu${}^{3+}$ line is at least as narrow as that of EuF${}_{3}$. Measurements of the variation of the absorption at different temperature enable the relative binding strengths of Eu${}^{2+}$ and Eu${}^{3+}$ to be determined. The binding for Eu${}^{2+}$ ($\Theta_{\mathrm{Debye}}=$ 145 K) was weaker than that of Eu${}^{3+}$ ($\Theta_{\mathrm{Debye}}=$ 261 K).

**Figure 8:**
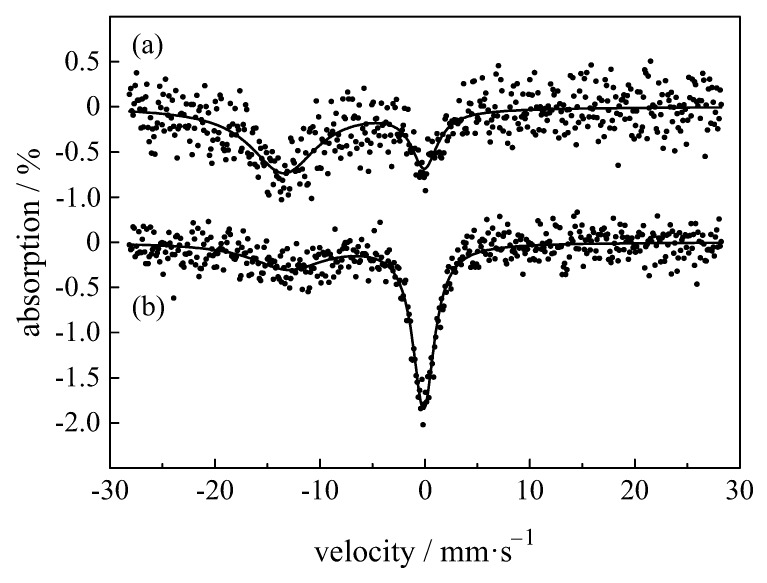
Mössbauer spectra of ZBLAN samples made with (**a**) 5.0 mol % EuCl${}_{2}$; and (**b**) 2.5 mol % EuCl${}_{2}$ and 2.5 mol % EuCl${}_{3}$ [91]. The solid lines represent a fit of the Mössbauer spectra using simple Lorentzian functions.

An important material in computed radiography is ZBLAN, which contains 51ZrF${}_{4}$·17BaF${}_{2}$·3.5LaF${}_{3}$·3AlF${}_{3}$·20NaF (values in mol %), which, when doped with Eu${}^{2+},$ has applications as storage phosphors [33]. Weber et al. [91] showed that EuCl${}_{3}$ heated at 710 ${}^{\circ }$C for 10 min resulted in almost equal amounts of EuCl${}_{2}$ and EuCl${}_{3}$. This could be important for production of EuCl${}_{2}$ storage phosphors since EuCl${}_{2}$ is more expensive than EuCl${}_{3}$. They also have shown that samples made with 5 mol % EuCl${}_{2}$ contained both EuCl${}_{2}$ and EuCl${}_{3}$ in the ratio 78:22 (Figure 8a). Samples made with a mixture of 2.5 mol % EuCl${}_{2}$ and 2.5 mol % EuCl${}_{3}$ had a ratio 37:63 (Figure 8b), i.e., 13% of the Eu${}^{2+}$ was oxidized. Johnson et al. [28] showed that EuCl${}_{2}$ as raw material oxidizes to Eu${}_{2}$O${}_{3}$ while EuCl${}_{2}$ in the glass oxidizes to EuCl${}_{3}$. Pfau et al. [94] studied ZBLAN doped with 0.5 mol % InF${}_{3}$ (to keep the ZrF${}_{4}$ from being reduced) and with BaCl${}_{2}$ and 5 mol % EuCl${}_{2}$ substituted for BaF${}_{2}$. About 88% of the europium was in the divalent state. When 5 mol % EuF${}_{2}$ was used, only 70% of the europium was in the divalent state. The isomer shift is not influenced by the thermal treatment of the glasses and amounts to approximately −14 mm/s. The Debye temperatures are $\Theta_{\mathrm{Debye}}=$ 147 K for Eu${}^{2+}$ and $\Theta_{\mathrm{Debye}}=$ 186 K for Eu${}^{3+}$.

### 4.3. Aluminates

BaMgAl${}_{10}$O${}_{17}$ (BAM) has the β-alumina structure and, when doped with Eu${}^{2+},$ it is a blue-emitting phosphor used for white light generation. It is generally unstable which is a problem for lighting applications. Fraknoy-Körös et al. [100] prepared Ba${}_{0.9}$Eu${}_{0.1}$MgAl${}_{10}$O${}_{17}$ in air and Mössbauer measurements show only trivalent Eu. In a reducing atmosphere both Eu${}^{3+}$ and Eu${}^{2+}$ are obtained. Without Mg and in a reducing atmosphere, mainly Eu${}^{2+}$ is produced. Boolchand et al. [98] investigated BAM containing different Eu concentrations of 6%, 12%, and 20%. The Mössbauer spectra were measured at 4.2 K and show significant magnetic hyperfine structure, though Boolchand interprets the spectra in terms of large quadrupole splitting. The isomer shifts are similar for all investigated Eu concentrations. Similar results are shown by Mishra et al. [130].

MAl${}_{12}$O${}_{19}$ (M = Ca, Sr, Ba) has the magnetoplumbite structure (related to β-alumina). The Mössbauer measurements of Hintzen et al. [101] also revealed a small amount of Eu${}^{3+}$ (<10%). The linewidth decreased with increasing Eu concentration from 11 mm/s to 3.6 mm/s, which is presumably due to unresolved paramagnetic hyperfine structure resulting from increased spin-spin relaxation. At a concentration of 25%, the emission spectrum shows an additional band at 490 nm as a consequence of the presence of very small traces of a second phase. No influence of the temperature on the spectra was observed in the range from 10 K to 300 K.

Arakawa et al. [95] reported an isomer shift of −9.7 mm/s which decreases with increasing size of the alkaline earth atom, a result of the decreased electron density at the Eu nuclei. The isomer shift decreases for increasing Eu concentration. The emission spectra of BaAl${}_{12}$O${}_{19}$ and CaAl${}_{12}$O${}_{19}$ contain one broad emission. For CaAl${}_{12}$O${}_{19},$ the peak shifts from 425 nm to 439 nm with an increase of Eu from 5% to 50% relative to Ca, while the peak of BaAl${}_{12}$O${}_{19}$ remains at approximately 450 nm. SrAl${}_{12}$O${}_{19}$:Eu${}^{2+}$ shows two emission peaks at 390 nm and 530 nm, for all investigated Eu concentrations.

Amorphous Al${}_{2}$O${}_{3}$ doped with Eu${}^{3+}$ shows a broad line, which was decomposed (not necessarily uniquely) into contributions from eight different quadrupole-split sites, as would be expected from an amorphous material [108]. The average isomer shift of 1.107 mm/s suggests strong covalent bonds and leads to the conclusion that Eu${}^{3+}$ ions replace Al${}^{3+}$ ions in the Al${}_{2}$O${}_{3}$ matrix.

Hölsä et al. [42] investigated CaAl${}_{2}$O${}_{4}$ with a low Eu concentration due to the segregation of the Eu ion from the CaAl${}_{2}$O${}_{4}$ phase. With Eu${}^{2+}$ doping, the compounds exhibit phosphorescence (persistent luminescence). Their Mössbauer spectra show a very broad line resulting from Eu${}^{2+}$ but also a small amount of Eu${}^{3+}$. EuAl${}_{2}$O${}_{4}$ has a green emission with the maximum at 515 nm [97]. Mössbauer spectra show two crystallographic sites for Eu${}^{2+}$ with different isomer shifts of −13.4 mm/s and −12.83 mm/s. The photoluminescence excitation spectrum shows two broad bands at 380 nm and 430 nm.

Tronc et al. [99] made measurements on LaMgAl${}_{11}$O${}_{19}$, which has the magnetoplumbite structure for compositions with 100% and 30% Eu replacing La using different methods of preparation. The spectra showed line broadening arising from paramagnetic hyperfine splitting, which was partially resolved in the most dilute (30% Eu) sample.

### 4.4. Oxide Glasses

Tanabe et al. [114] found that in silicate and aluminate glasses the isomer shift of Eu${}^{3+}$ increases with increasing optical basicity of the glasses. The optical basicity measures the electron donation by the oxygen anions to the metal ion used as a probe (Eu), the correlation indicates that the charge transferred to the Eu${}^{3+}$ ion occupies mainly 6s shells and hence the phosphates have the smallest charge in the 6s shells compared to the other oxide glasses.

**Figure 9:**
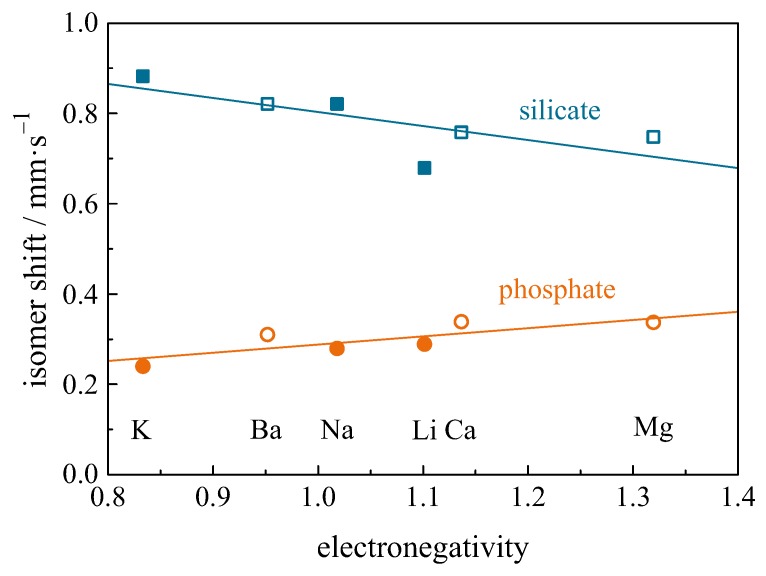
Relationship between isomer shift and the electronegativity of network-modifying alkali (full symbols) and alkali earths (open symbols) in silicate (squares) and phosphate (circles) glasses [112].

In silicate glasses M${}_{3}$Na${}_{8}$Si${}_{13}$O${}_{65}$ (M = Mg, Ca, Ba) the Eu${}^{3+}$ isomer shift decreases with decreasing modifier cation size, i.e., increasing electronegativity (Figure 9) [112]. The shift is close to that of Eu${}_{2}$O${}_{3}$ [110], suggesting that Eu${}^{3+}$ ions locate in sites similar to those in Eu${}_{2}$O${}_{3}$. Musić et al. [111] obtained similar values for the isomer shift of Eu${}^{3+}$ in sodium borosilicate glasses.

In related aluminosilicate crystal Ba${}_{0.95}$Eu${}_{0.05}$Al${}_{2}$Si${}_{2}$O${}_{8}$, the europium is mostly Eu${}^{2+}$ with an isomer shift of −14 mm/s. About 5 to 10% of the europium is Eu${}^{3+}$ with an isomer shift close to 0 mm/s [45].

In metaphosphate glasses M${}_{5}$Al${}_{4}$P${}_{25}$O${}_{76}$ (M = Mg, Ca, Ba), the Eu${}^{3+}$ isomer shift increases with decreasing modifier cation size, i.e., increasing electronegativity. This is in contrast to the behaviour in silicate glasses [112] and is ascribed to the effect of the short π-bonds between the modifier atoms and the non-bridging phosphate chains. Metaphosphate glasses containing Zn, Sr, and Pb were investigated by Concas et al. [115]. The glass containing Pb has a lower isomer shift compared to the other two samples. A sodium phosphate glass containing Eu${}^{3+}$ and Ce${}^{3+}$ was irradiated by multipulse excimer-UV-laser irradiation, leading to an extinction of the luminescence [113]. The as-made glass showed an isomer shift of approximately 0.79 mm/s and decreases to 0.37 mm/s after irradiation. It is believed that the electronic traps created by irradiation expand the wave function of 5s electrons, thus decreasing the electronic density at the nucleus.

The borate glasses B${}_{2}$O${}_{3}$:Eu and B${}_{2}$O${}_{3}$-A${}_{2}$O${}_{3}$:Eu (A = Li, Na, K) were studied by Winterer et al. [102] for Eu concentrations between 0.1% and 33%. The isomer shifts of −13.0 mm/s corresponded to Eu${}^{2+}$. The more dilute glasses showed paramagnetic hyperfine splitting with fields of 35 T. The more concentrated ones showed quadrupole broadened Eu${}^{2+}$ lines, and also some Eu${}^{3+}$. Fujita et al. [36] obtained similar results for B${}_{2}$O${}_{3}$-Na${}_{2}$O:Eu glasses. In sodium borate glass prepared under a reducing atmosphere, Fujita [131] observed the Eu${}^{2+}$ and Eu${}^{3+}$ absorption bands in the Mössbauer spectra and investigated spectral hole burning in Eu${}^{3+}$ emission spectra. The relative hole area increases with the absorption area ratio, *R*, of Eu${}^{2+}$/Eu${}^{3+}$ in the Mössbauer spectrum up to a value of R=0.3 and decreases slightly for larger ratios.

Fujita et al. [36] studied 15EuO·85((1−x)B${}_{2}$O${}_{3}$·*x*Na${}_{2}$O) and found that the Eu${}^{2+}$ isomer shifts increase as the concentration of Na${}_{2}$O increases and decrease as the concentration of EuO increases. In borate glasses with increasing network modifier content, the amount of three-coordinated boron decreases and that of four-coordinated boron increases up to about 33 mol % Na${}_{2}$O content, beyond which the four-coordinated boron starts to decrease (boron anomaly) due to the appearance of non-bridging oxygen. Hence, the electron density at the Eu nucleus drastically increases in this compositional region, since the non-bridging oxygen has more electron donation ability than the bridging oxygen and increases the 6s electron density effectively.

Nemov et al. [126] investigated germanium glasses (BaGeO${}_{3}$)${}_{1-x-y}$(Al${}_{2}$O${}_{3}$)${}_{x}$(0.45CaF${}_{2}$·0.55MgF${}_{2}$)${}_{y}$ with Eu${}_{2}$O${}_{3}$ concentrations varying from 5 mol % to 20 mol %. Europium was observed only in the trivalent state. The influence of the Eu content was minimal, while the increase of the fluorine content led to a larger isomer shift as well as to a higher frequency (shorter wavelength) of the PL emission band. The linewidth of both the isomer shift and the emission band decreased for increasing fluorine content.

### 4.5. Lanthanides and Related Compounds Sc_2_O_3_, Y_2_O_3_, In_2_O_3_

The crystal structures of the lanthanide sesquioxides Ln${}_{2}$O${}_{3}$ are complicated: cubic, monoclinic, and hexagonal structures have been observed. For cubic Eu${}_{2}$O${}_{3}$, shift values are close to 1.0 mm/s [36,117,132,133,134]. For Eu${}^{3+}$ in lanthanide oxides and the related compounds Sc${}_{2}$O${}_{3}$, Y${}_{2}$O${}_{3}$, and In${}_{2}$O${}_{3}$ the isomer shifts increase in the sequence Gd${}_{2}$O${}_{3}$, Y${}_{2}$O${}_{3}$, Sc${}_{2}$O${}_{3}$, Lu${}_{2}$O${}_{3}$, and In${}_{2}$O${}_{3}$, while the mean Ln-O distance decreases in the same sequence (Table 3) [66,119]. The monoclinic system also has two sites for the Ln ions, while the hexagonal system has only one site. Hintzen et al. [66] compared monoclinic and cubic Eu${}_{2}$O${}_{3}$ and obtained slightly larger isomer shifts for the monoclinic structure.

**Figure 10:**
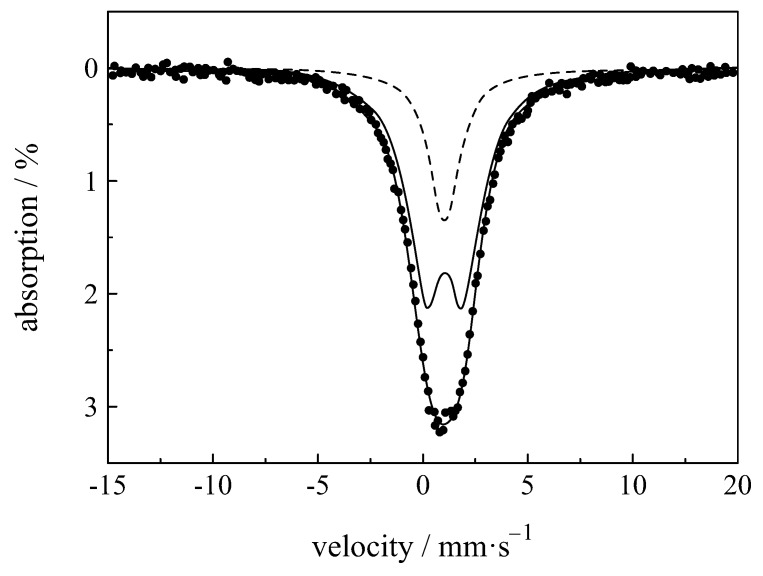
Mossbauer spectra of nanocrystalline Sc${}_{1.8}$Eu${}_{0.2}$O${}_{3}$. The experimental data (dots) and the fit curve (full line) are shown along with the components associated to the $C_{3i}$ and $C_{2}$ sites (dashed line and full line, respectively) [117].

The spectra of Sc${}_{2}$O${}_{3}$:Eu${}^{3+}$ show a clear decomposition into two lines (Figure 10) [66,117]. Concas et al. [117] investigated cubic Sc${}_{1.8}$Eu${}_{0.2}$O${}_{3}$ and found isomer shifts of 1.18 and 1.38 mm/s for the sites $C_{3i}$ and $C_{2}$, respectively. Hintzen et al. [66] obtained a higher difference between the isomer shifts of the two different sites for cubic Sc${}_{1.9}$Eu${}_{0.1}$O${}_{3}$ of 0.7 and 2.59 mm/s.

Bohus et al. [67] and Kuzmann et al. [116] investigated monodisperse Y${}_{2}$O${}_{3}$:Eu${}^{3+}$ spherical nanoparticles as well as Y${}_{2}$O${}_{3}$@Eu${}^{3+}$ core-shell structures. The core-shell nanoparticles had an isomer shift of 1.03 mm/s, which agrees with that found for pure Eu${}_{2}$O${}_{3}$ and is independent of the Eu${}^{3+}$ doping level. Other papers gave similar results for Y${}_{2}$O${}_{3}$:Eu${}^{3+}$ [66,100,117].

**Figure 11:**
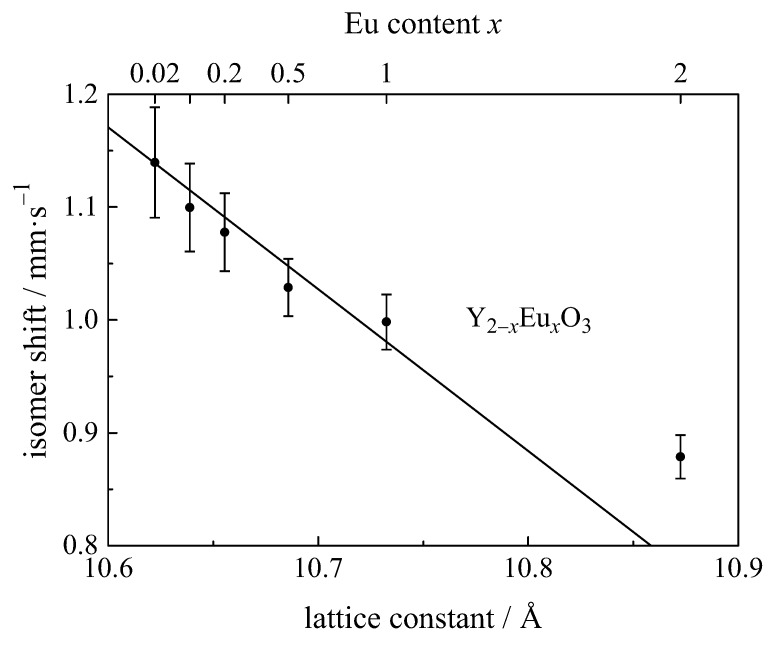
The average isomer shift as a function of the measured lattice parameter, *a*, for a series of cubic Y${}_{2-x}$Eu${}_{x}$O${}_{3}$ samples. The solid line indicates the volume dependence [66].

Y${}_{2-x}$Eu${}_{x}$WO${}_{6}$ (x= 0.05–0.4) was investigated by van Noort and Pompa [123]. The Eu ions were in the trivalent state and occupied three different sites, two sites with symmetry $C_{2}$ and one site with symmetry $C_{1}$. Small differences in covalency produce the isomer shifts obtained of 1.25 mm/s, 0.65 mm/s, and 0.05 mm/s, respectively, and which increased with Eu${}^{3+}$ concentration, due to an increase of the lattice parameter *a* (Figure 11).

Monoclinic and cubic Gd${}_{1.9}$Eu${}_{0.1}$O${}_{3}$ showed different average isomer shifts of 1.08 and 1.05 mm/s, respectively, resulting from the different oxygen surroundings of the Eu${}^{3+}$ ions in the two crystallographic structures [66,86].

### 4.6. Vanadates

Bibicu et al. [120] observed two broad lines in the Mössbauer spectrum of YVO${}_{4}$:Eu${}^{3+}$ nanocrystals doped with 5 at.% showing the presence of Eu${}^{3+}$ ions in different possible micro-environments. Fraknoy-Körös et al. [100] found similar results. Li et al. [121] investigated EuVO${}_{4}$ prepared at two different temperatures resulting in different phases, which were identified as scheelite and zircon. The scheelite phase showed a strong luminescence. Stadtnik et al. [105] investigated three different vanadate compounds: Eu${}_{2}$VO${}_{4}$, Eu${}_{3}$V${}_{2}$O${}_{7}$, and Eu${}_{0.6}$Sr${}_{0.4}$VO${}_{2.96}$. The first two compounds showed Eu${}^{2+}$ and Eu${}^{3+}$, while the latter showed only Eu${}^{3+}$. The amount of Eu${}^{2+}$ was found to be 39% and 17% and had quadrupole interactions of −18.0 mm/s and −13.2 mm/s for Eu${}_{2}$VO${}_{4}$ and Eu${}_{3}$V${}_{2}$O${}_{7}$, respectively. Divalent and trivalent Eu ions are found to exist simultaneously at the same crystallographic sites in Eu${}_{2}$VO${}_{4}$ and Eu${}_{3}$V${}_{2}$O${}_{7}$.

### 4.7. Titanates

TiO${}_{2}$:Eu amorphous powder showed only Eu${}^{3+}$ sites with isomer shifts of approximately 0.5 mm/s independent of Eu concentration, while the linewidth increases with increasing Eu concentration [125]. Calcination at temperatures above 500 ${}^{\circ }$C lead to the formation of brookite and anatase phases, while for temperatures higher than 1000 ${}^{\circ }$C pure rutile phase was formed. The Mössbauer spectra for all temperatures were similar, the luminescence intensity decreases with increasing calcination temperature.

However, Ningthoujam et al. [106] reported on a TiO${}_{2}$:Eu anatase phase, which showed two Mössbauer absorption bands, corresponding to Eu${}^{3+}$ and Eu${}^{2+}$ with isomer shifts of −0.62 mm/s and −13.04 mm/s, respectively. The samples were annealed at 500 ${}^{\circ }$C and 900 ${}^{\circ }$C and the spectra showed one peak corresponding to Eu${}^{3+}$ with isomer shifts of −0.48 mm/s and −0.64 mm/s, respectively. In the 900 ${}^{\circ }$C annealed sample, the Eu${}_{2}$Ti${}_{2}$O${}_{7}$ phase was formed, which did not show any luminescence. Annealing of the sample at temperatures from 300 ${}^{\circ }$C to 1000 ${}^{\circ }$C did not change the isomer shift significantly.

Ti${}_{2}$O${}_{7}$ (cubic) pyrochlores fluoresce below room temperature [87]. The Mössbauer spectra show Eu${}^{3+}$ with a large quadrupole interaction of −19 mm/s and a pronounced anisotropic *f*-factor (Goldanski-Karyagin Effect). The isomer shift is 0.8 mm/s.

### 4.8. Nitrides

Europium nitrodosilicate Eu${}_{2}$SiN${}_{3}$ has mixed valence and its Mössbauer spectrum contains lines from both Eu${}^{2+}$ and Eu${}^{3+}$ [50]. For GaN:Eu, Mössbauer spectra showed that the Eu was preferentially in the trivalent state [135]. EuSi${}_{2}$O${}_{2}$N${}_{2}$ showed an emission in the yellow spectral range and the Mössbauer spectra showed only Eu${}^{2+}$ at 78 K [52].

### 4.9. Sulfides

CaS has a rock-salt structure and Eu${}^{2+}$ is supposed to replace Ca${}^{2+}$ in the lattice. For CaS:Eu luminophors, Danilkin et al. [136] observed only one Mössbauer absorption peak, corresponding to Eu${}^{3+}$ ions. Pham-Thi et al. [103] synthesized CaS:Eu using the flux method with Na and K. The Mössbauer spectrum of the sample prepared with Na polysulphide flux showed only Eu${}^{3+}$ absorption while with K flux both ions were detected.

### 4.10. ZrO_2_

Mössbauer spectra of ZrO${}_{2}$:Eu doped with 1 and 2 mol % Eu${}_{2}$O${}_{3}$ showed only Eu${}^{3+}$ with an isomer shift independent of Eu concentrations, while the linewidth decreases with increasing Eu concentration indicating that the europium environment is changing [127]. It was concluded that, at low concentrations, europium ions occupy sites in both the tetragonal and monoclinic structure and with increasing Eu concentration the incorporated ions are mainly substituted into the tetragonal structure substituting Zr${}^{4+}$.

### 4.11. Yttrium Aluminum Garnet (YAG:Eu)

Constantinescu et al. [81] used Mössbauer spectroscopy to investigate structural changes during the phase transition from amorphous to crystalline in yttrium aluminum garnet (Y${}_{3}$Al${}_{5}$O${}_{12}$). Annealing the YAG with temperatures lower than the phase transition temperature, which is between 900 ${}^{\circ }$C and 915 ${}^{\circ }$C [137], revealed two peaks in the Mössbauer spectra, while annealing with higher temperatures gave one transmission peak. For annealing at temperatures from 930 ${}^{\circ }$C to 1400 ${}^{\circ }$C, an increase in crystalline size was obtained from X-ray diffraction measurements and a decrease in photoluminescence linewidths resulting from the higher crystallinity as well as a slight increase in Mössbauer absorption area was observed for all investigated temperatures [81].

### 4.12. Discussion

#### 4.12.1. Correlation of Isomer Shift with Bond Length and Covalency

**Figure 12:**
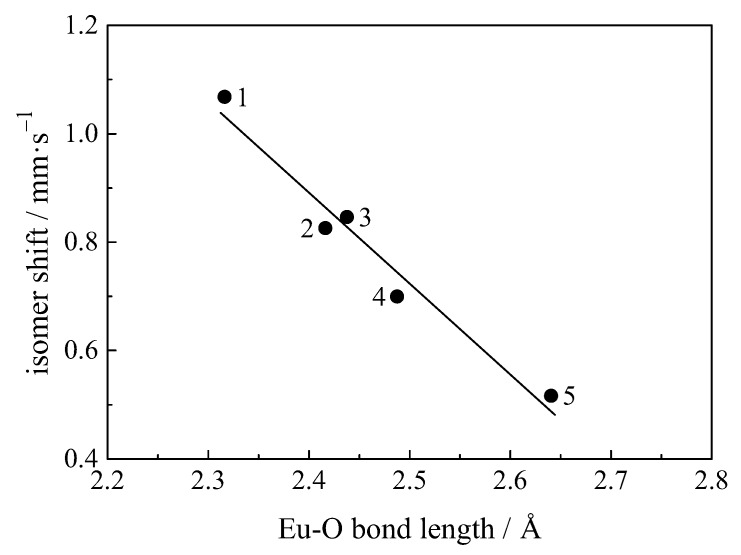
Relationship between the isomer shift and the Eu–O bond length in some oxide crystals: **1**—c-type Eu${}_{2}$O${}_{3}$ (6); **2**—EuBa${}_{2}$Cu${}_{3}$O${}_{7}$ (8); **3**—Eu${}_{3}$Ga${}_{5}$O${}_{12}$ garnet (8); **4**—EuAl${}_{3}$(BO${}_{3}$)${}_{4}$ huntite (9); **5**—EuAlO${}_{3}$ perovskite (12). The coordination number of Eu${}^{3+}$ ion is shown in parentheses [114].

An increase in isomer shift corresponds to an increase in covalency (see Table 1). It is seen that for luminescent materials there is a correlation between the isomer shift and the bond length Eu–X (Figure 12), since an increase in the bond length decreases the local density at the europium sites and hence the electron density at the Eu nuclei. Such a correlation has been found for Eu${}^{3+}$ in Y${}_{2-x}$Eu${}_{x}$O${}_{3}$ Hintzen (Figure 11) [66,119], Eu${}^{3+}$ in Ln${}_{2}$O${}_{3}$ compounds [66,86], Y${}_{2}$O${}_{3}$:Eu${}^{3+}$ nanoparticles and Y${}_{2}$O${}_{3}$@Eu${}^{3+}$ core-shell structures [67,116], Eu${}^{2+}$ in oxides, sulphides and selenides [138].

Related correlations also exist between the isomer shift and europium concentration, which result from the difference in size between the europium and host ions e.g., Eu${}^{2+}$ and Eu${}^{3+}$ in La${}_{1-x}$Eu${}_{x}$MgAl${}_{11}$O${}_{19}$ [99] and Eu${}^{2+}$ fluorogermanate glasses (Nemov et al. [126].)

Glass correlations are found between the isomer shift and the electronegatively (or size) of the modifying cations in sodium silicate M${}_{3}$Na${}_{8}$Si${}_{13}$O${}_{33}$ and aluminophosphate M${}_{5}$Al${}_{4}$P${}_{25}$O${}_{76}$ glasses (M = Mg, Ca, Sr), as shown in Figure 9 [112].

#### 4.12.2. Determination of Site Occupancies

The occupancy of each site when there are several sites in a material may be determined since there are small differences between the isomer shift of them by computer fitting the spectrum. The unresolved and overlapping contributions from each site leads to a broadened spectrum.

The lanthanide sesquioxides with the general formula Ln${}_{2}$O${}_{3}$ have complicated crystal structures. In the cubic system, there are two different sites for the lanthanide ions: 25% in a more symmetric site $C_{3i}$ (S${}_{6}$) and 75% in a less symmetric site $C_{2}$, which shows quadrupole broadening [66]. For Sc${}_{2}$O${}_{3}$:Eu and In${}_{2}$O${}_{3}$:Eu the two sites could be resolved [66,86]. Their average isomer shifts are 1.27 mm/s and 1.38 mm/s respectively. For Sc${}_{1.9}$Eu${}_{0.1}$O${}_{3}$ the shifts for the sites $C_{3i}$ and $C_{2}$ are 0.70 mm/s and 2.59 mm/s, respectively [66]. In other compounds, the difference in shift is less and is determined from the (slightly asymmetrical) line broadening. Hintzen et al. [66] showed that the difference in isomer shift between the $C_{3i}$ and $C_{2}$ sites increases linearly with decreasing lattice parameter *a*. The decrease of the Eu–O distance increases the electron density, which affects the $C_{3i}$ site more than the $C_{2}$ site, as shown in Figure 10 [66,117]. Concas et al. [86,119] obtained in cubic nanocrystalline Y${}_{2}$O${}_{3}$ an occupational probability of the $C_{3i}$ and $C_{2}$ sites of 27% and 73%, respectively compared with bulk material values of 22% ($C_{3i}$) and 78% ($C_{2}$) [119]. Concas et al. [118] also investigated Lu${}_{1.8}$Eu${}_{0.2}$O${}_{3}$. They prepared the samples in four different ways: (1) combustion with urea; (2) combustion with urea and sintered; (3) combustion with glycine; and (4) combustion with glycine and sintered. All samples show an isomer shift of 1.25 mm/s averaged over the two different sites $C_{3i}$ and $C_{2}$. The samples show a variation in site occupancy with the different preparation methods. For the nanocrystalline powders (1) and (3), approximately 20–23% of the Eu ions are in $C_{3i}$ symmetry, while for the ceramics only 15–16% of the Eu ions are in $C_{3i}$ symmetry. Thus, there is preferential occupation of the $C_{2}$ site, especially for the (spherical) ceramic samples, which causes a higher fluorescence intensity since the ${}^{5}$D${}_{0}$ to ${}^{7}$F${}_{2}$ transition is forbidden. In monoclinic Gd${}_{2}$O${}_{3}$, the Eu${}^{3+}$ ions are equally distributed over the three different crystallographic sites [66].

In Y${}_{2-x}$Eu${}_{x}$WO${}_{6}$:Eu, the Eu${}^{3+}$ ions occupy three different sites, two with $C_{2}$ and one with $C_{1}$ symmetry. Small differences in covalency produce the isomer shifts obtained of 1.25 mm/s, 0.65 mm/s, and 0.05 mm/s, respectively and which increased with Eu${}^{3+}$ concentration [123].

BaCa${}_{2}$Y${}_{6}$O${}_{12}$:Eu${}^{3+}$ has two different sites with isomer shifts of 0.2 and 1.5 mm/s. At the site with the lower shift, Eu${}^{3+}$ replaces Y${}^{3+}$and for the higher Eu${}^{2+}$ replaces Ca${}^{2+}$. The Y${}^{3+}$ site is preferred with respect to the Ca${}^{2+}$ site by about a factor of 2 [53].

## 5. Conclusions

We have reviewed the use of Mössbauer spectroscopy for characterizing luminescent materials activated by europium. Mössbauer spectroscopy is a powerful probe of europium as it is element-specific and can provide knowledge of the valence state, covalency, site symmetry, and occupation and coordination number, all of which may be important for the study and development of luminescent materials. The large difference in isomer shift between Eu${}^{2+}$ and Eu${}^{3+}$ enables the ionic state to be identified and the relative amounts of each to be determined in a host material. The spectra confirm that europium usually substitutes as Eu${}^{2+}$ for a divalent ion like Ca${}^{2+}$ and as Eu${}^{3+}$ for a trivalent ion like Al${}^{3+}$ or Y${}^{3+}$, but that both oxidation states may sometimes occur together since Eu${}^{2+}$ easily oxidizes to Eu${}^{3+}$. The shift also depends upon the ligand X since it affects the electron density at the europium nuclei. Roughly, it scales with the local density, and so increases with increasing coordination number and decreasing Eu–X bond length or ligand diameters. This means it increases for strong covalent bonding (or low electronegativity) and for higher europium concentrations. The quadrupole splitting gives information about local symmetry and can be important for identifying or confirming the crystal site. Mössbauer spectra can also distinguish between crystallographic sites with the same ionic state and measure site occupancy in unusual circumstances, for example, in nanoparticles. Photoluminescence spectroscopy can clearly distinguish that Eu${}^{2+}$ and Eu${}^{3+}$: Eu${}^{2+}$ are characterized by a broad emission, while Eu${}^{3+}$ shows narrow emission lines, but, as the spectra overlap, it cannot give a quantitative estimate of the relative amounts of each. In summary, Mössbauer spectroscopy is a unique and leading technique in the characterization of materials containing multi-valent elements with distinctly different isomer shifts.

## Figures and Tables

**Table 1:** Data for Mössbauer isomer shifts of Eu in binary compounds. Data from [11].

Compound with Divalent Eu	Isomer Shift in mm/s	Compound with Trivalent Eu	Isomer Shift in mm/s
EuF${}_{2}$	−13.58	EuF${}_{3}$	0 (standard)
EuBr${}_{2}$	−13.43	EuCl${}_{3}$	0.05
EuCl${}_{2}$	−13.10	EuBr${}_{3}$	0.17
EuI${}_{2}$	−12.50	EuI${}_{3}$	0.29
EuO	−12.28	Eu${}_{2}$O${}_{3}$	1.01

**Table 2:** Data for isomer shifts of Eu${}^{2+}$ at room temperature with respect to EuF${}_{3}$ in different hosts. U—broad unresolved magnetic hyperfine structure, P—partially resolved magnetic hyperfine structure.

Host	Isomer Shift in mm/s	Hyperfine Structure for Diluted Eu${}^{2+}$	References
ZBLAN and Cl glasses	−13	U	[91]
CaF${}_{2}$	−13.4	hyperfine field of 34 T	[92]
ZrF${}_{4}$·BaF${}_{2}$·EuF${}_{2}$·ThF${}_{4}$ glass	−14.18 ${}^{\left(g\right)}$	U	[93]
ZBLAN glass	−14.3 ${}^{\left(d\right)}$	U	[94]
Sr${}_{0.95}$Eu${}_{0.05}$Al${}_{12}$O${}_{19}$	−9.7		[95]
Ba${}_{0.95}$Eu${}_{0.05}$Al${}_{12}$O${}_{19}$	−10.1		[95]
Ca${}_{0.5}$Eu${}_{0.5}$Al${}_{12}$O${}_{19}$	−11.2		[95]
Ca${}_{0.95}$Eu${}_{0.05}$Al${}_{12}$O${}_{19}$	−11.7		[95,96]
Eu${}_{2}$AlO${}_{3.75}$N${}_{0.1}$	−11.98 ${}^{\left(e\right)}$		[97]
BaMgAl${}_{10}$O${}_{17}$:Eu	−12 ${}^{\left(f\right)}$		[3]
BaMgAl${}_{10}$O${}_{17}$:6%Eu	−12.55 ${}^{\left(a,h,c\right)}$		[98]
EuAl${}_{2}$O${}_{4}$	−13.1 ${}^{\left(a\right)}$		[97]
EuMgAl${}_{11}$O${}_{19}$	−13.14	P	[99]
Ba${}_{0.5}$Eu${}_{0.5}$MgAl${}_{11}$	−13.4		[100]
Lu${}_{0.7}$Eu${}_{0.3}$MgAl${}_{11}$O${}_{19}$	−13.47	P	[99]
BaAl${}_{2}$Si${}_{2}$O${}_{8}$	−14 ${}^{\left(f\right)}$	U	[45]
Sr${}_{0.75}$Eu${}_{0.25}$Al${}_{12}$O${}_{19}$	−14.2 ${}^{\left(h\right)}$	U	[101]
B${}_{2}$O${}_{3}$ glass	−13 ${}^{\left(a\right)}$	hyperfine field of 35 T	[102]
(0.7B${}_{2}$O${}_{3}$·0.3Na${}_{2}$O)${}_{0.85}$·(EuO)${}_{0.15}$ glass	−13.15	P	[36]
(0.7B${}_{2}$O${}_{3}$·0.3Na${}_{2}$O)${}_{0.80}$·(EuO)${}_{0.20}$ glass	−13.15	P	[36]
(0.7B${}_{2}$O${}_{3}$·0.3Na${}_{2}$O)${}_{0.70}$·(EuO)${}_{0.30}$ glass	−13.48	P	[36]
(0.9B${}_{2}$O${}_{3}$·0.1Na${}_{2}$O)${}_{0.85}$·(EuO)${}_{0.15}$ glass	−13.55	P	[36]
Eu${}_{2}$SiN${}_{3}$	−10.66	U	[50]
CaS:Eu	−11.5		[103]
EuS	−11.7		[104]
Eu${}_{2}$VO${}_{4}$	−11.8		[105]
TiO${}_{2}$:Eu	−12.04 ${}^{\left(b\right)}$		[106]
EuSi${}_{2}$O${}_{2}$N${}_{2}$	−12.3 ${}^{\left(f\right)}$		[52]
Eu${}_{3}$V${}_{2}$O${}_{7}$	−12.5		[105]

${}^{\left(a\right)}$ average over different sites; ${}^{\left(b\right)}$ converted from Eu${}_{2}$O${}_{3}$; ${}^{\left(c\right)}$ 4.2 K; ${}^{\left(d\right)}$ 6 K; ${}^{\left(e\right)}$ 20 K; ${}^{\left(f\right)}$ 77/78 K; ${}^{\left(g\right)}$ increasing with decreasing temperature; ${}^{\left(h\right)}$ decreasing with increasing Eu concentration.

**Table 3:** Data for isomer shifts of Eu${}^{3+}$ at room temperature with respect to EuF${}_{3}$ in different hosts.

Host	Isomer Shift in mm/s	References
ZrF${}_{4}$·BaF${}_{2}$·EuF${}_{2}$·ThF${}_{4}$ glass	0.06 ${}^{\left(g\right)}$	[93]
ZBLAN glass	0 ${}^{\left(d\right)}$	[94]
ZBLAN and Cl glasses	0	[91]
(45−x)AlF${}_{3}$·*x*AlPO${}_{4}$·5EuF${}_{3}$·30CaF${}_{2}$·20BaF${}_{2}$	−0.05	[107]
Al${}_{1.93}$Eu${}_{0.07}$O${}_{3}$ (mainly amorphous)	1.11	[108]
Eu${}_{2}$AlO${}_{3.75}$N${}_{0.1}$	0.28 ${}^{\left(e\right)}$	[97]
Eu${}_{0.1}$Al${}_{0.9}$PO	0.22	[109]
La${}_{0.7}$Eu${}_{0.3}$MgAl${}_{11}$O${}_{19}$	−0.28	[99]
Sr${}_{0.75}$Eu${}_{0.25}$Al${}_{12}$O${}_{19}$	−0.3 ${}^{\left(h\right)}$	[101]
LaMgAl${}_{11}$O${}_{19}$	−0.35	[99]
BaMgAl${}_{10}$O${}_{17}$	−0.93 ${}^{\left(c\right)}$	[98]
SiO${}_{2}$·Na${}_{2}$O·BaO·ZnO·Eu${}_{2}$O${}_{3}$	1.07 ${}^{\left(b\right)}$	[110]
SiO${}_{2}$·B${}_{2}$O${}_{3}$·Na${}_{2}$O·Eu${}_{2}$O${}_{3}$	1	[111]
BaO·SiO${}_{2}$	0.82	[112]
50Na${}_{2}$O·44P${}_{2}$O${}_{5}$·3Eu${}_{2}$O${}_{3}$·3CeO${}_{2}$	0.79 ${}^{\left(b\right)}$	[113]
CaO·SiO${}_{2}$	0.76	[112]
50SiO${}_{2}$·25A1${}_{2}$0${}_{3}$·25Eu${}_{2}$O${}_{3}$	0.46	[114]
(4ZnO)${}_{0.975}$·3B${}_{2}$O${}_{3}$·0.025Eu${}_{2}$O${}_{3}$	0.45	[115]
0.1Eu(PO${}_{3}$)${}_{3}$·0.9Zn(PO${}_{3}$)${}_{2}$	0.37	[115]
0.1Eu(PO${}_{3}$)${}_{3}$·0.9Sr(PO${}_{3}$)${}_{2}$	0.36	[115]
CaO·P${}_{2}$O${}_{5}$	0.36	[112]
BaO·P${}_{2}$O${}_{5}$	0.32	[112]
0.1Eu(PO${}_{3}$)${}_{3}$·0.9Pb(PO${}_{3}$)${}_{2}$	0.29	[115]
(0.7B${}_{2}$O${}_{3}$·0.3Na${}_{2}$O)${}_{1-x}$·(EuO)${}_{x}$	0	[36]
In${}_{1.9}$Eu${}_{0.1}$O${}_{3}$	1.38 ${}^{\left(a\right)}$	[66]
Lu${}_{1.9}$Eu${}_{0.1}$O${}_{3}$	1.35 ${}^{\left(a\right)}$	[66]
Y${}_{2}$O${}_{3}$:Eu${}^{3+}$ (nanoparticle)	1.28 ${}^{\left(h\right)}$	[67,116]
Sc${}_{1.8}$Eu${}_{0.2}$O${}_{3}$	1.28 ${}^{\left(a\right)}$	[117]
Sc${}_{1.9}$Eu${}_{0.1}$O${}_{3}$	1.27 ${}^{\left(a\right)}$	[66]
Lu${}_{1.8}$Eu${}_{0.2}$O${}_{3}$	1.25 ${}^{\left(a\right)}$/1.23 ${}^{\left(a\right)}$	[117,118]
La${}_{1.9}$Eu${}_{0.1}$O${}_{3}$	1.2	[66]
Y${}_{1.8}$Eu${}_{0.2}$O${}_{3}$	1.2	[100]
Y${}_{1.8}$Eu${}_{0.2}$O${}_{3}$	1.14 ${}^{\left(a\right)}$	[117]
Y${}_{1.9}$Eu${}_{0.1}$O${}_{3}$	1.12 ${}^{\left(a\right)}$	[66]
Gd${}_{1.9}$Eu${}_{0.1}$O${}_{3}$ (monoclinic)	1.08 ${}^{\left(a\right)}$	[66]
Gd${}_{1.9}$Eu${}_{0.1}$O${}_{3}$ (cubic)	1.05 ${}^{\left(a\right)}$	[66]
Y${}_{2}$O${}_{3}$@Eu${}^{3+}$ (core-shell)	1.03	[67,116]
Gd${}_{1.8}$Eu${}_{0.2}$O${}_{3}$ (nanocrystalline)	0.97	[86,119]
Eu${}_{3}$V${}_{2}$O${}_{7}$	0.6	[105]
YVO${}_{4}$ (nanoparticle)	0.5 ${}^{\left(b\right)}$	[120]
Eu${}_{2}$VO${}_{4}$	0.5 ${}^{\left(a\right)}$	[105]
Y${}_{0.963}$Eu${}_{0.037}$VO${}_{4}$	0.49	[100]
EuVO${}_{4}$ (zircon)	−0.06	[121]
EuVO${}_{4}$ (scheelite)	−0.55	[121]
YAG:Eu nanocrystal	1.47 ${}^{\left(b\right)}$	[81]
BaCa${}_{2}$Y${}_{6}$O${}_{12}$:Eu	1.0 ${}^{\left(a\right)}$	[53]
Eu${}_{2}$SiN${}_{3}$	0.85	[50]
Eu${}_{2}$Ti${}_{2}$O${}_{7}$	0.8 ${}^{\left(b\right)}$	[87]
KY${}_{0.735}$Eu${}_{0.265}$W${}_{2}$O${}_{8}$	0.68 ${}^{\left(f\right)}$	[122]
Y${}_{1.95}$Eu${}_{0.05}$WO${}_{6}$	0.65 ${}^{\left(a,i\right)}$	[123]
CuLa${}_{0.99}$Eu${}_{0.01}$O${}_{2}$	0.61 ${}^{\left(i\right)}$	[124]
6 TiO${}_{2}$:Eu	0.6 ${}^{\left(h\right)}$	[125]
(BaGeO${}_{3}$)${}_{1-x-y}$·(Al${}_{2}$O${}_{3}$)${}_{x}$·(0.45CaF${}_{2}$·0.55MgF${}_{2}$)${}_{y}$	0.48	[126]
TiO${}_{2}$:Eu	0.38 ${}^{\left(b\right)}$	[106]
ZrO${}_{2}$:Eu	0.3	[127]
CaS:Eu	−0.8	[103]

${}^{\left(a\right)}$ average over different sites; ${}^{\left(b\right)}$ converted from Eu${}_{2}$O${}_{3}$; ${}^{\left(c\right)}$ 4.2 K; ${}^{\left(d\right)}$ 6 K; ${}^{\left(e\right)}$ 20 K; ${}^{\left(f\right)}$ 77/78 K; ${}^{\left(g\right)}$ increasing with decreasing temperature; ${}^{\left(h\right)}$ decreasing with increasing Eu concentration; ${}^{\left(i\right)}$ increasing with increasing Eu concentration.
